# The intelligent networks for double-diffusion and MHD analysis of thin film flow over a stretched surface

**DOI:** 10.1038/s41598-021-97458-2

**Published:** 2021-09-28

**Authors:** Iftikhar Uddin, Ikram Ullah, Muhammad Asif Zahoor Raja, Muhammad Shoaib, Saeed Islam, M. S. Zobaer, K. S. Nisar, C. Ahamed Saleel, Saad Alshahrani

**Affiliations:** 1 Department of Mathematics, Abdul Wali Khan University Mardan (KP), Mardan, 23200 Pakistan; 2 Department of Sciences and Humanities, National University of Computer and Emerging Sciences Peshawar Campus, KP, Peshawar, 25000 Pakistan; 3 Future Technology Research Center, National Yunlin University of Science and Technology, 123 University Road, Section 3, Douliou, Yunlin 64002 Taiwan, R.O.C.; 4 Department of Mathematics, COMSATS University Islamabad, Attock Campus, Attock, 43600 Pakistan; 5 McGovern Medical School, The University of Texas Health Science Center at Houston, Houston, TX USA; 6 Department of Mathematics, College of Arts and Sciences, Prince Sattam Bin Abdulaziz University, Wadi Aldawaser, 11991 Saudi Arabia; 7 Department of Mechanical Engineering, College of Engineering, King Khalid University, Asir-Abha, 61421 Saudi Arabia

**Keywords:** Nanoscale materials, Fluid dynamics, Computational science

## Abstract

This study presents a novel application of soft-computing through intelligent, neural networks backpropagated by Levenberg–Marquardt scheme (NNs-BLMS) to solve the mathematical model of unsteady thin film flow of magnetized Maxwell fluid with thermo-diffusion effects and chemical reaction (TFFMFTDECR) over a horizontal rotating disk. The expression for thermophoretic velocity is accounted. Energy expression is deliberated with the addition of non-uniform heat source. The PDEs of mathematical model of TFFMFTDECR are transformed to ODEs by the application of similarity transformations. A dataset is generated through Adams method for the proposed NNs-BLMS in case of various scenarios of TFFMFTDECR model by variation of rotation parameter, magnetic parameter, space dependent heat sink/source parameter, temperature dependent heat sink/source parameter and chemical reaction controlling parameter. The designed computational solver NNs-BLMS is implemented by performing training, testing and validation for the solution of TFFMFTDECR system for different variants. Variation of various physical parameters are designed via plots and explain in details. It is depicted that thin film thickness increases for higher values of disk rotation parameter, while it diminishes for higher magnetic parameter. Furthermore, higher values of Dufour number and the corresponding diminishing values of Soret number causes enhancement in fluid temperature profile. Further the effectiveness of NNs-BLMS is validated by comparing the results of the proposed solver and the standard solution of TFFMFTDECR model through error analyses, histogram representations and regression analyses.

Thin film flow can be expressed as thin liquid layer flowing over substrate or surface due to gravity or shear stresses (the external forces) and the holding free boundary is called free surface. Free surface is that one, at which, the parallel shear stresses vanish. Liquid’s free surface is that one where it is in contact with the air. The applications of thin film liquid flows have very wide range uses in the mass and heat transfer devises, printing technology, spinning disks reactors, drop spreading processes, microelectronic industry, lubricating gears, distillation columns. Moreover, coating or spin coating (thin film flow) is necessary for improvement of durability, cooling or heat transfer process and work efficiency. Emslie et al.^1^ initiated the work on liquid film flow in research field, by considering the flow over a rotating disk. The stated work was extended by Jenekhe^2^ and Flack et al. ^3^ by including heat and mass transfer effects and taking non-Newtonian fluids for analysis. Washo^4^ performed experimental findings of spin coating problem. Nath and Kumari^5^ carried out research work on thin film unsteady flow with disk rotation effect. Thin film flow along with various effects, like activation energy, radiation, Joule heating, nanomaterials, unsteadiness, thin film thickness Brownian motion and thermophoresis, over rotating disk were studied by various researchers^6–11^. The impact of double-diffusion and flow of thin film liquid over rotating surface have been extensively analyzed by many researchers^12–18^.

The artificial intelligent based neural networks (NNs) is an effective and authentic solver, which has been used extensively in diversified fields of science. A brief survey is provided here, which provides the effectiveness of NNs technique. For instance, the effective applicability can be seen with applications include nonlinear HIV infection with CD4+ T-cells based mathematical model^19^, the model of Maxwell nanofluid thin film flow over a rotating and stretched surface^20^, the integrated system of power plant including stochastic wind^21^, nonlinear dynamics system of COVID-19^22^, the model of squeezing flow^23^, singular nonlinear pantograph differential models^24^, the piezostage actuator Bouc–Wen hysteresis system^25^. All the above-mentioned facts imply that the artificial intelligent base techniques have a variety of applications in sundry fields of science and technology. The application of computational intelligent neural networks processes can be helpful for the solution nonlinear systems of thin nanoliquid film flow model of Maxwell fluid with thermo-diffusion effect and chemical reaction (TFFMFTHECR) over rotating disk.

The notable features and motivations of this analysis are highlighted as:

- A novel design of intelligent, neural networks backpropagated by Levenberg–Marquardt scheme (NNs-BLMS) is incorporated for numerical interpretations of TFFMFTHECR mode.
- The system of PDEs of TFFMFTDECR model are transformed to system of ODEs by the use of suitable similarity transformations.
- A dataset is generated through Adams method for the proposed NNs-BLMS in case of sundry scenarios of TFFMFTDECR model by varying rotation parameter, magnetic parameter, space dependent heat source/sink parameter, temperature dependent heat source/sink parameter and chemical reaction parameter.
- The efficiency and performance of NNs-BLMS is verified by comparing the results with the standard solution of TFFMFTDECR model through analyses of error, graphical outcomes of histograms and regression.
- The outcomes depicted that thin film thickness increases for higher values of disk rotation parameter, while it diminishes for higher magnetic parameter. Furthermore, higher values of Dufour number and the corresponding diminishing values of Soret number causes enhancement in fluid temperature profile.

The rest of the article is presented as: the problem TFFMFTDECR model formulation is given in “Mathematical model formulation and interpretation of the outcomes” section, the results of NNs-BLMS in case of involved parameters are elaborated in “Solution methodology and results” section, “conclusion” of the work is placed in the very last.

## Mathematical model formulation and interpretation of the outcomes

**Figure 1:**
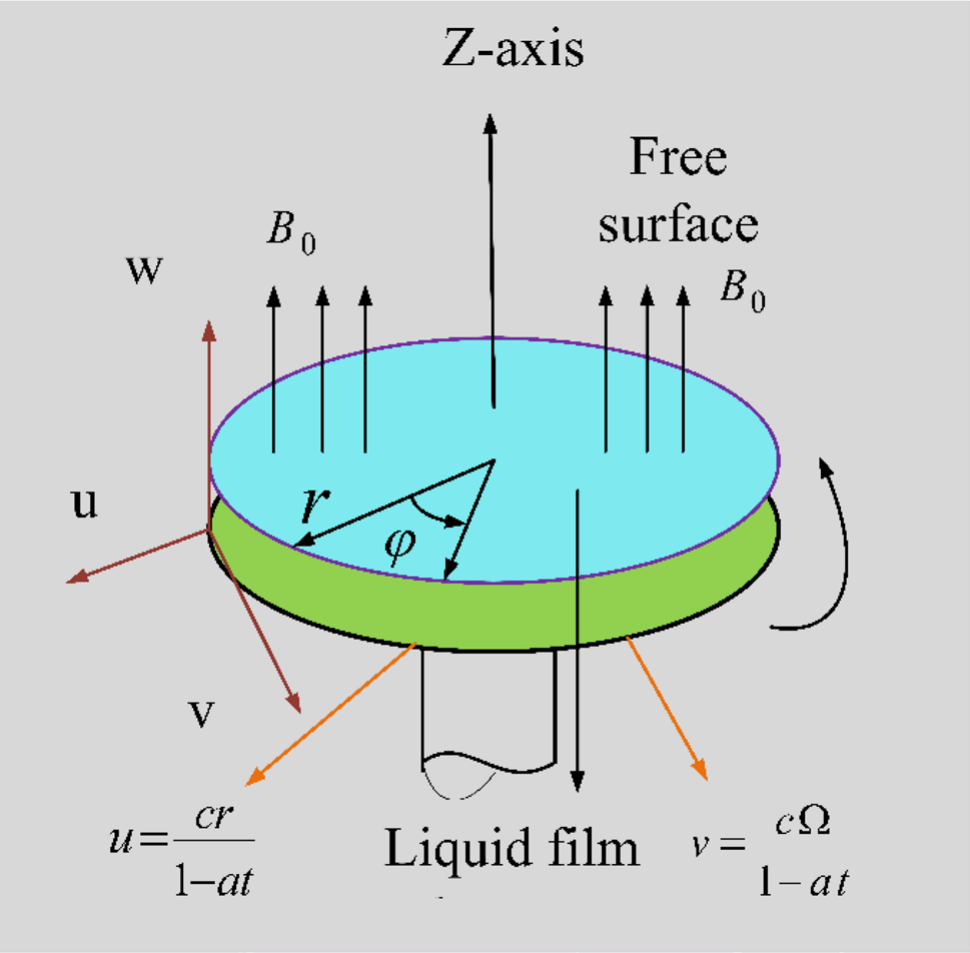
The geometry of the transient thin film flow problem.

The Maxwell fluid thin film unsteady MHD^26–28^ flow over a rotating stretchable disk in cylindrical coordinate system $(r,\;\varphi ,\;z)$ with Soret and Dufour effects is considered. A first order chemical reaction is included. The liquid thin film flow is due to rotation and stretching behavior of the disk having velocities $\left( {u,v} \right) = \left( {r\;c\;/(1 - a\;t),\;\Omega \;r\;/\;(1 - a\;t)} \right)$ in $(r,\;\;\phi )$ directions, where $\Omega$ is rotation rate, c is stretching rate and $a > 0$ is a constant as shown in Fig. 1. The thickness of the Maxwell nanofluid film over rotating and stretching disk is $h( r, t)$. The disk surface concentration and temperature are mathematically defined as: $\left( {C_{S} ,\;T_{S} } \right)$ = $\left( {C_{0} - C_{ref} \;\left( {r^{2} \;\Omega /\left( {\upsilon \;\left( {1 - a\;t} \right)^{3/2} } \right)} \right),\;T_{0} - T_{ref} \;\left( {r^{2} \;\Omega /\left( {\upsilon \;\left( {1 - a\;t} \right)^{3/2} } \right)} \right)} \right)$, where concentration and temperature at origin are $\left( {C_{0} ,\;T_{0} } \right)$, while the reference constant concentration and temperature are $\left( {C_{ref} ,\;\;T_{ref} } \right)$. The flow is influenced by magnetic field $B_{0} /\sqrt {1 - a\;t}$ in the *z*-direction. The mathematical representation of the above-mentioned physical problem is given by the governing equations^29,30^ as:

1 $$\frac{\partial }{\partial r}\left( u \right) + \;\frac{u}{r}\; + \;\frac{\partial }{\partial z}\left( w \right)\;\; = \;\;0,$$

2 $$\frac{\partial }{\partial t}\left( u \right) + u\frac{\partial }{\partial r}\left( u \right) + w\frac{\partial }{\partial z}\left( u \right) - \frac{{v^{2} }}{r} + \lambda_{1} \left( \frac{{\partial^{2} }}{{\partial t^{2} }}\left( u \right) + u^{2} \frac{{\partial^{2} }}{{\partial r^{2} }}\left( u \right) + 2u\frac{{\partial^{2} }}{\partial t\partial r}\left( u \right) + 2w\frac{{\partial^{2} }}{\partial t\partial z}\left( u \right) - \frac{2vu}{r}\frac{\partial }{\partial r}\left( v \right) + w^{2} \frac{{\partial^{2} }}{{\partial z^{2} }}\left( u \right) + \frac{{v^{2} u}}{{r^{2} }} + 2wu\frac{{\partial^{2} }}{\partial z\partial r}\left( u \right) - \frac{2v}{r}\frac{\partial }{\partial t}\left( u \right) + \frac{{v^{2} }}{r}\frac{\partial }{\partial r}\left( u \right) - \frac{2wv}{r}\frac{\partial }{\partial z}\left( v \right) \right) = \left( {\frac{{\partial^{2} }}{{\partial z^{2} }}\left( u \right)} \right)\;\upsilon - \;\frac{{B_{0}^{2} }}{{\left( {1 - t\;a} \right)}}\frac{\sigma }{\rho }\;\left( {u + \lambda_{1} \;\left( {\frac{\partial }{\partial t}\;\left( u \right) + w\;\frac{\partial }{\partial z}\;\left( u \right)} \right)\;} \right),$$

3 $$\frac{\partial }{\partial t}\;\left( {\;v\;} \right)\; + \;u\;\frac{\partial }{\partial r}\;\left( {\;v\;} \right)\; + \;w\;\frac{\partial }{\partial z}\;\left( {\;v\;} \right) + \frac{vu}{r} + \lambda_{1} \left( \frac{{\partial^{2} }}{{\partial t^{2} }}\left( v \right) + 2u\frac{{\partial^{2} }}{\partial t\partial r}\left( v \right) + \frac{2v}{r}\frac{\partial }{\partial t}\left( u \right)\;\; + \;\;u^{2} \frac{{\partial^{2} }}{{\partial r^{2} }}\left( v \right) + 2w\frac{{\partial^{2} }}{\partial t\partial z}\left( v \right) + w^{2} \frac{{\partial^{2} }}{{\partial z^{2} }}\left( v \right) - \frac{{u^{2} v}}{{r^{2} }} + \frac{2vu}{r}\frac{\partial }{\partial r}\left( u \right) + 2wu\frac{{\partial^{2} }}{\partial z\partial r}\left( v \right) + \frac{2wv}{r}\frac{\partial }{\partial z}\left( u \right) + \frac{{v^{2} }}{r}\frac{\partial }{\partial r}\left( v \right) - \frac{{v^{3} }}{{r^{2} }} \right) = \left( {\frac{{\partial^{2} }}{{\partial z^{2} }}\left( v \right)} \right)\;\upsilon - \;\frac{{B_{0}^{2} }}{{\left( {1 - t\;a} \right)}}\;\frac{\sigma }{\rho }\;\left( {v + \left( {\frac{\partial }{\partial t}\;\;\left( v \right)\; + \;w\;\;\frac{\partial }{\partial z}\;\left( v \right)} \right)\;\;\lambda_{1} } \right),$$

4 $$\frac{\partial }{\partial t}\;\left( T \right)\; + \;u\frac{\partial }{\partial r}\;\left( T \right)\; + w\frac{\partial }{\partial z}\;\left( T \right) = \frac{k}{{\rho c_{p} }}\frac{{\partial^{2} }}{{\partial z^{2} }}\;\left( T \right) + \frac{q^{\prime\prime\prime}}{{\rho \;c_{p} }} + \frac{{K_{T} \;D_{B} }}{{c_{p} c_{s} }}\frac{{\partial^{2} }}{{\partial z^{2} }}\;\left( {C\;} \right),$$

5 $$\frac{\partial }{\partial t}\left( C \right) + u\frac{\partial }{\partial r}\left( C \right) + w\frac{\partial }{\partial z}\left( C \right) = \frac{{K_{T} \;D_{B} }}{{T_{m} }}\frac{{\partial^{2} }}{{\partial z^{2} }}\left( T \right) - \frac{{\partial \left( {\left( {C - C_{0} } \right)\;\;V_{T} } \right)}}{\partial z} + D_{B} \frac{{\partial^{2} }}{{\partial z^{2} }}\left( C \right) - kr\;\left( {C - C_{0} } \right),$$

where non-uniform, space and temperature dependent, heat source/sink $q^{\prime\prime\prime}$^31^, thermophoretic velocity $V_{T}$^32^ and thermophoretic coefficient $k_{V}$ are given by:

6 $$q^{\prime\prime\prime} = \frac{k\;u\left( r \right)}{{r\;\upsilon }}\;\left\{ {A\;F^{\prime}\;\left( {T_{s} - T_{0} } \right)\; + \;\left( {T - T_{0} } \right)\;B} \right\},$$

7 $$V_{T} = \frac{{ - \;\upsilon \;k_{V} \;}}{{T_{ref} }}\;\frac{\partial T}{{\partial z}},\,\,k_{V} \; = \;\frac{{2\;c_{b} \;\left( {\lambda_{g} /\lambda_{p} \; + \;k_{n} \;c_{1} \;} \right)\;\left( {c_{1} \; + \;\;c_{2} \;e^{{ - c_{3} /k_{n} }} } \right)}}{{\left( {1\; + \;3\;k_{n} \;c_{m} } \right)\;\left( {1\; + \;2\;\lambda_{g} /\;\lambda_{p} \; + \;2k_{n} \;c_{1} } \right)}}.$$

The boundary conditions for the thin film flow of Maxwell nanofluid problem are:

8 $$\begin{aligned} & w = 0,\;\;u = \frac{r\;c}{{1 - at}},\;\;v = \frac{\Omega \;r}{{1 - at}},\;\;\;C = C_{s} ,\;\;T = T_{s} \;\;\;\;{\text{at}}\;\;z = 0, \\ & w = u\frac{\partial }{\partial r}\left( h \right) + \frac{\partial }{\partial t}\left( h \right),\;\;\frac{\partial }{\partial z}\left( u \right) = \frac{\partial }{\partial z}\left( v \right) = \;\frac{\partial }{\partial z}\left( C \right) = \frac{\partial }{\partial z}\left( T \right) = 0\;\;{\text{at}}\;z = h. \\ \end{aligned}$$

where the triplet $$\left( {u,\;v,\;w} \right)$$ represents the velocity components corresponding to $$\left( {r,\;\varphi ,\;z} \right)$$. The expressions $$\left( {\rho ,\;k,\;T,\;C,\;\lambda_{1} ,\;c_{p} ,\;T_{S} ,\;C_{S} ,\;K_{T} ,\;D_{B} \;,\;kr,\;\upsilon ,} \right.\;c_{s} ,\;$$$$\left. {\;T_{0,} \;C_{0} ,\;A,\;\;B,\;\;k,\;\;T_{m} } \right)$$ appearing in the above equations are fluid density, thermal conductivity, temperature, concentration, time relaxation parameter, specific heat at constant pressure, temperature at surface, concentration at surface, thermal diffusion, diffusion coefficient for species, chemical reaction coefficient, kinematic viscosity, concentration susceptibility, temperature at origin, concentration at origin, space dependent heat source/sink, temperature dependent heat source/sink, thermal conductivity of fluid and fluid mean temperature, respectively. The positive values of $$A$$ and $$\;\;B$$ i.e. $$\;\left( {A\;\;\left( + \right)} \right)$$ and $$\;\left( {B\;\;\left( + \right)} \right)$$ corresponds to internal heat generation, while negative values of these parameters i.e. $$\;\left( {A\;\;\left( - \right)} \right)$$ and $$\;\left( {B\;\;\left( - \right)} \right)$$ represents absorption parameters, respectively.

The similarity variables are defined as:

9 $$\begin{aligned} & w = - 2\left( {\frac{\Omega \;\upsilon }{{1 - t\;a}}} \right)^{\frac{1}{2}} \;\;F\left( \eta \right),\;u = \frac{\Omega \;r}{{1 - t\;a}}F^{{\prime }} \left( \eta \right),\;\;v = \frac{\Omega \;r}{{1 - t\;a}}G\left( \eta \right),\;\;\eta = \left( {\frac{\Omega \;}{{\upsilon \;\left( {1 - t\;a} \right)}}} \right)^{\frac{1}{2}} \;\;z, \\ & T = T_{0} - T_{ref} \frac{{r^{2} \;\Omega \;}}{{\left( {1 - t\;a} \right)^{\frac{3}{2}} \;\upsilon }}\theta \left( \eta \right),\;\;h\left( t \right) = \left( {1 - t\;a} \right)^{\frac{1}{2}} \left( {\frac{\upsilon }{\Omega }} \right)^{\frac{1}{2}} \;\beta ,\; \\ & C = C_{0} - C_{ref} \frac{{r^{2} \;\Omega }}{{\left( {1 - t\;a} \right)^{\frac{3}{2}} \;\upsilon }}\phi \left( \eta \right). \\ \end{aligned}$$

Substituting Eq. (9) into Eqs. (1)–(5) and Eq. (8) the following system of ODEs with corresponding boundary conditions are obtained:

10 $$F^{\prime\prime\prime} - S\left( {\frac{\eta }{2}F^{\prime\prime} + F^{\prime}} \right) - \left( {F^{{\prime}{2}} - G^{2} - 2\;F^{\prime\prime}F} \right) - \;\beta_{1} (\;S^{2} \left( {\frac{1}{4}\eta^{2} F^{\prime\prime\prime} + 2F^{\prime} + \frac{7}{4}\eta F^{\prime\prime}} \right) + S\left( {2F^{{\prime}{2}} - 2G^{2} - 6F^{\prime\prime}F} \right) + S\eta \left( {F^{\prime\prime}F^{\prime} - G^{\prime}G - 2F^{\prime\prime\prime}F} \right) + 4F^{\prime\prime\prime}F^{2} - 4FG^{\prime}G - 4F^{\prime\prime}F^{\prime}F\;) - M\left( {\beta_{1} \left( {S\;F^{\prime} + \frac{1}{2}S\;\eta \;F^{\prime\prime} - 2F^{\prime\prime}\;F} \right) + \;F^{\prime}} \right) = 0,$$

11 $$G^{\prime\prime} - S\left( {G + \frac{\eta }{2}\;G^{\prime}} \right) - 2\left( {F^{\prime}G - G^{\prime}F} \right) - \;\beta_{1} (\;S^{2} \left( {\frac{1}{4}\;\eta^{2} \;G^{\prime\prime} + \frac{7}{4}\eta \;G^{\prime} + 2\;G} \right) + S\left( {4\;F^{\prime}\;G - 6\;F\;G^{\prime}} \right) + S\eta \left( {F^{\prime}\;G^{\prime} + F^{\prime\prime}\;G - 2\;G^{\prime\prime}F} \right) - 4F^{\prime\prime}\;G\;F - 4F^{\prime}\;G^{\prime}\;F + 4\;G^{\prime\prime}\;F^{2} \;) - M\left( {\beta_{1} \left( {S\;G - 2\;F\;G^{\prime} + \frac{1}{2}\eta \;G^{\prime}\;S} \right) + G} \right) = 0,$$

12 $$\frac{1}{\Pr }\;\theta ^{\prime\prime}\; + \;Du\;\phi ^{\prime\prime}\; + \;2\left( {\theta ^{\prime}F - \;\theta F^{\prime}} \right)\; - \frac{1}{2}Sc\left( {3\;\theta \; + \;\eta \;\theta ^{\prime}} \right) + \frac{1}{\Pr }\left( {B\;\theta \; + A\;F^{\prime}} \right) = 0,$$

13 $$\phi ^{\prime\prime} - \;Sc\;K_{R} \;\phi - Sc\;\tau \;\left( {\phi ^{\prime}\;\theta ^{\prime}\; + \phi \;\theta ^{\prime\prime}} \right) + \;Sc\;Sr\;\theta ^{\prime\prime}\; + 2\;Sc\;\left( {\phi ^{\prime}\;F\; - \;\phi \;F^{\prime}} \right)\; - \frac{Sc}{2}\left( {\eta \;\phi ^{\prime}\; + \;3\;\phi } \right) = 0,$$

14 $$\begin{aligned} & F\left( 0 \right) = 0,\;\;F^{\prime}\left( 0 \right) = \omega ,\;\;G\left( 0 \right) = 1,\;\;\theta \left( 0 \right) = 1,\;\;\phi \left( 0 \right) = 1, \hfill \\ & F\left( \beta \right) = \frac{S\beta }{4},\;\;F^{\prime\prime}\left( \beta \right) = G^{\prime}\left( \beta \right) = \theta \left( \beta \right) = \phi ^{\prime}\left( \beta \right) = 0. \hfill \\ \end{aligned}$$

The physical parameters appearing in above equations are given by:

15 $$\left. \begin{gathered} \;Du = \frac{{K_{T} \;D_{B} \;\left( {C_{S} - C_{0} } \right)}}{{c_{s} \;\upsilon \;c_{p} \;\left( {T_{S} - T_{0} } \right)}},\;\;\;Sr = \frac{{K_{T} \;D_{B} \;\left( {T_{S} - T_{0} } \right)}}{{\upsilon \;T_{m} \;\left( {C_{S} - C_{0} } \right)}},\;\;\;\tau = - \frac{{k_{V} \;\left( {T_{S} - T_{0} } \right)}}{{T_{ref} }},\;\;\; \hfill \\ \Pr = \frac{\upsilon }{\alpha },\;M = \frac{{\sigma B_{0}^{2} }}{\rho \Omega },\;K_{R} = \frac{r\;(kr)}{{u_{w} }},\;Sc = \frac{\upsilon }{{D_{B} }},\;S = \frac{a}{\Omega },\;\omega = \frac{c}{\Omega },\;\beta_{1} = \frac{{\Omega \;\lambda_{1} }}{1 - at}. \hfill \\ \end{gathered} \right\}$$

where $S$ is unsteadiness parameter, $M$ is Magnetic parameter, $\beta_{1}$ is Deborah number, $\Pr$ is Prandtl number, $Du$ is Dufour number, $\tau$ is thermophoretic number, $K_{R}$ is chemical reaction parameter, $Sr$ is Soret number, $Sc$ is Schmidt number and $\omega$ is rotation parameter.

The value of parameter $\beta$ represents $\eta$ at free surface. Therefore, Eq. (9) gives:

16 $$\beta = \;h\;\left( {\frac{\Omega }{{\left( {1 - t\;a} \right)\upsilon }}} \right)^{\frac{1}{2}} .$$

The local Nusselt number, which represents heat transfer rate and Sherwood number, which is mass transfer rate are given by:

17 $$Nu_{r} = \frac{{r\;q_{w} }}{{k\;\left( {T_{S} - T_{0} } \right)}}\;,\;\;Sh_{r} = \frac{{r\;j_{w} }}{{\left( {C_{s} - C_{0} } \right)\;k}},\;q_{w} \; = \; - k\;\left( {\frac{\partial T}{{\partial z}}} \right)_{z = 0} \;,\;j_{w} \; = \; - k\;\left( {\frac{\partial C}{{\partial z}}} \right)_{z = 0} \;.$$

The expressions in dimensionless form of these quantities are:

18 $${\text{Re}}^{{ - \frac{1}{2}}} Nu_{r} = - \;\theta ^{\prime}\left( 0 \right),\;\;\;{\text{Re}}^{{ - \frac{1}{2}}} \;Sh_{r} = - \;\phi ^{\prime}\left( 0 \right),\;\;$$

where, ${\text{Re}}^{\frac{1}{2}} = r\;\left( {\Omega /\left( {\left( {1 - t\;a} \right)\;\upsilon } \right)} \right)^{1/2}$ stands for local Reynolds number.

## Solution methodology and results

**Figure 2:**
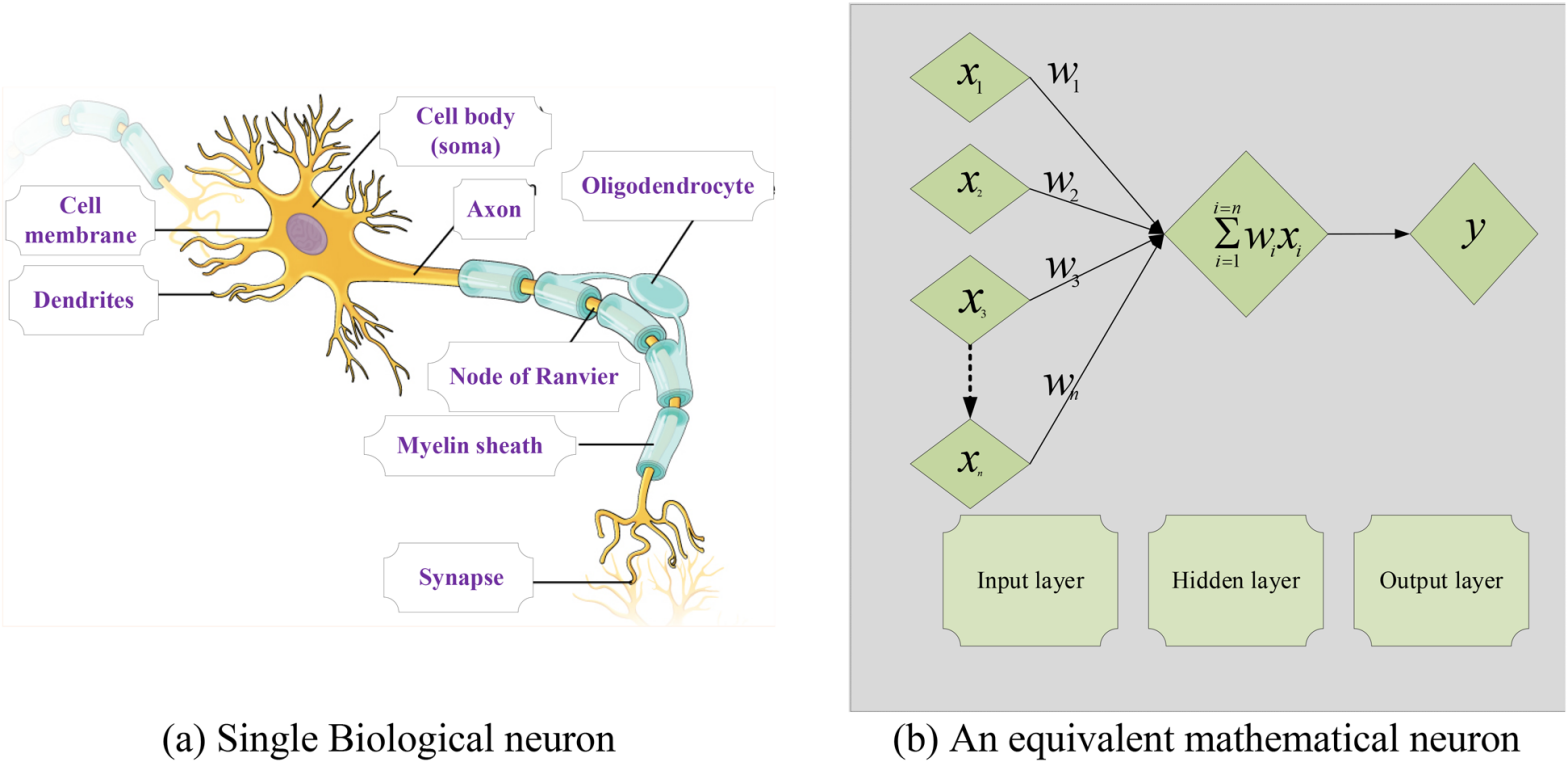
A single neural structure.

**Figure 3:**
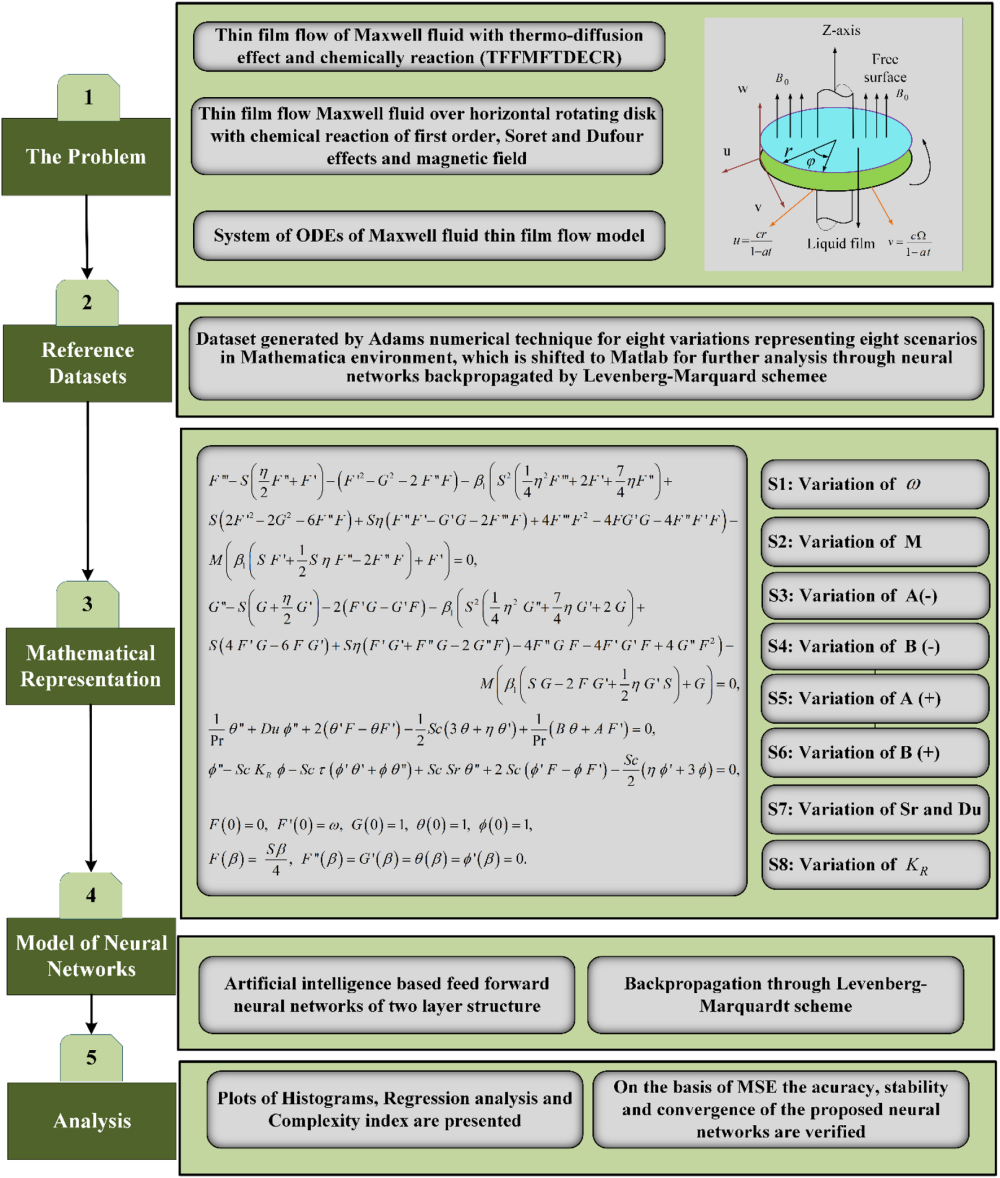
Work flow diagram of NNs- BLMS for TFFMFTDECR.

The application of computational numerical solver, intelligent neural networks backpropagated by Levenberg–Marquardt scheme (NNs-BLMS), is applied to numerically investigate thin film flow of Maxwell fluid with thermo-diffusion effects TFFMFTDECR model. The solver NNs-BLMS is applied to process the dataset generated by Adams numerical technique. The process of neural networks is presented in the form of single biological neuron as well as mathematical equivalent structure is given in Fig. 2. The overall process of this study is presented as single flow chart in Fig. 3. Eight variants of TFFMFTDECR model are processed through ‘nftool’, using NNs-BLMS for the solution.

The TFFMFTDECR model, which is given in Eqs. (10)–(14) is treated numerically for eight variations $\omega$, $M$, $A\;( - )$, $B\;( - )$, $A\;( + )$, $B\;( + )$, $(Sr,\;Du)$ and $K_{R}$, where each variation represents a scenario. The eight scenarios are presented in detail with the help of Table 1 along with three cases for each scenario, while Table 2 represents the detail of non-varying parameters during the analysis.

**Table 1:** Illustration of eight scenarios (1–8) along with cases (1–3) for TFFMFTDECR model.

Scenario	Case	Description of variation of physical parameters									
		$\beta$	$\omega$	M	Variation of $A$ and $B$ for positive and negative values				Sr	Du	$K_{R}$
					$A( - )$	$B( - )$	$A( + )$	$B( + )$			
1	1	2.21	1.0	1.0	–	–	0.2	0.2	0.2	0.2	0.3
	2	2.57	1.3								
	3	3.14	1.9								
2	1	2.73	1.0	0.0	–	–	0.2	0.2	0.2	0.2	0.3
	2	2.21		1.0							
	3	1.70		3.0							
3	1	2.21	1.0	1.0	− 0.5	0.1	0.2	0.2	0.2	0.2	0.3
	2	2.21			− 1.5						
	3	2.21			− 2.0						
4	1	2.21	1.0	1.0	–	− 0.5	0.2	0.2	0.2	0.2	0.3
	2	2.21				− 1.5					
	3	2.21				− 2.0					
5	1	2.21	1.0	1.0	–	0.1	0.5	0.2	0.2	0.2	0.3
	2	2.21					1.5				
	3	2.21					2.0				
6	1	2.21	1.0	1.0	–	–	1.0	0.5	0.2	0.2	0.3
	2	2.21						1.5			
	3	2.21						2.0			
7	1	2.21	1.0	1.0	–	–	0.2	0.2	0.07	1.0	0.3
	2	2.21							0.4	0.175	
	3	2.21							1.0	0.07	
8	1	2.21	1.0	1.0	–	–	0.2	0.2	0.2	0.2	0.0
	2	2.21									0.3
	3	2.21									0.9

**Table 2:** Description of physical quantities, which are non-varying during the study.

$S$	$\beta_{1}$	$\Pr$	$Sc$	$\tau$
1.0	0.6	1.7	1.0	0.9

**Figure 4:**
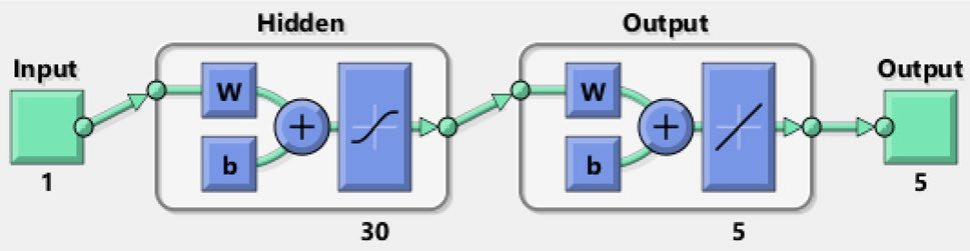
Architecture of neural networks.

The ‘NDSolve^33–36^^’^ tool is used in Mathematica environment to solve the system of ODEs of TFFMFTDECR for eight variations as described in Table 1. After generating the numerical reference dataset through Adams technique, it was exported to Matlab (Matlab 2021a, with Licence 40727596, and URL is as: https://www.mathworks.com/products/matlab.html) environment NNs-BLMS. The inputs used for cases (1–3) of scenarios (1–8) for step size 0.01, the detail is given in Table 3. The input values are distributed as 70%, 15%, and 15% for train, validation and testing, respectively. The designed NNs-BLMS with of two layered structure is given in Fig. 4.

**Table 3:** Illustration of eight scenarios (1–8) along with cases (1–3) for TFFMFTDECR model.

Scenario	Case	$\beta$	$\omega$		Starting value		Step size		End value		Input Values in dataset for NNs-BLMS
1 ($\omega$)	1	2.21	1.0		0		0.01		2.21		222
	2	2.57	1.3		0		0.01		2.57		258
	3	3.14	1.9		0		0.01		3.14		315
			$$M$$								
2 ($M$)	1	2.73	0.0		0		0.01		2.73		274
	2	2.21	1.0		0		0.01		2.21		222
	3	1.70	3.0		0		0.01		1.70		171
			$$A( - )$$								
3 ($A( - )$)	1	2.21	− 0.5		0		0.01		2.21		222
	2	2.21	− 1.5		0		0.01		2.21		222
	3	2.21	− 2.0		0		0.01		2.21		222
			$$B( - )$$								
4 ($B( - )$)	1	2.21	− 0.5		0		0.01		2.21		222
	2	2.21	− 1.5		0		0.01		2.21		222
	3	2.21	− 2.0		0		0.01		2.21		222
			$$A( + )$$								
5 ($A( + )$)	1	2.21	0.5		0		0.01		2.21		222
	2	2.21	1.5		0		0.01		2.21		222
	3	2.21	2.0		0		0.01		2.21		222
			$$B( + )$$								
6 ($B( + )$)	1	2.21	0.5		0		0.01		2.21		222
	2	2.21	1.5		0		0.01		2.21		222
	3	2.21	2.0		0		0.01		2.21		222
			$Sr$	$Du$							
7 (*Sr*, *Du*)	1	2.21	0.07	1.0	0		0.01		2.21		222
	2	2.21	0.4	0.175	0		0.01		2.21		222
	3	2.21	1.0	0.07	0		0.01		2.21		222
			$$K_{R}$$								
8 ($K_{R}$)	1	2.21	0.0		0		0.01		2.21		222
	2	2.21	0.3		0		0.01		2.21		222
	3	2.21	0.9		0		0.01		2.21		222

**Figure 5:**
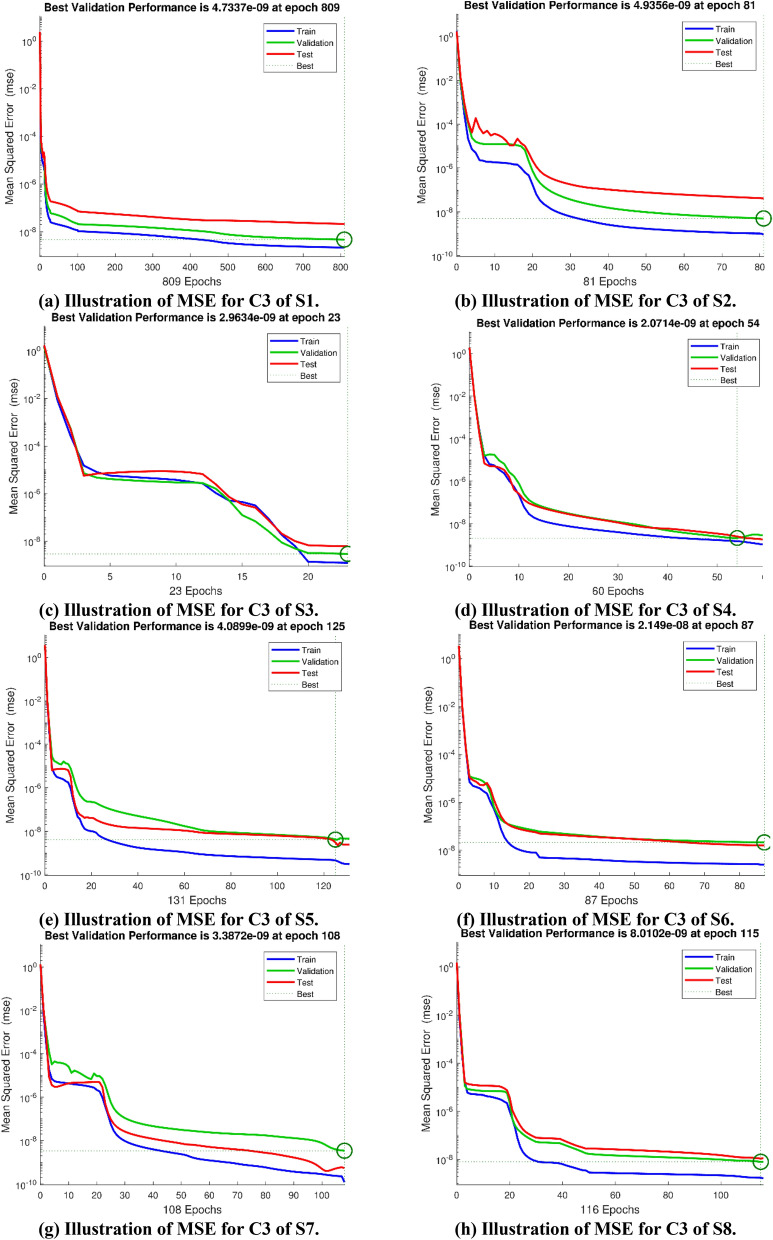
Performance illustration through graphs of C3 of all eight scenarios.

**Figure 6:**
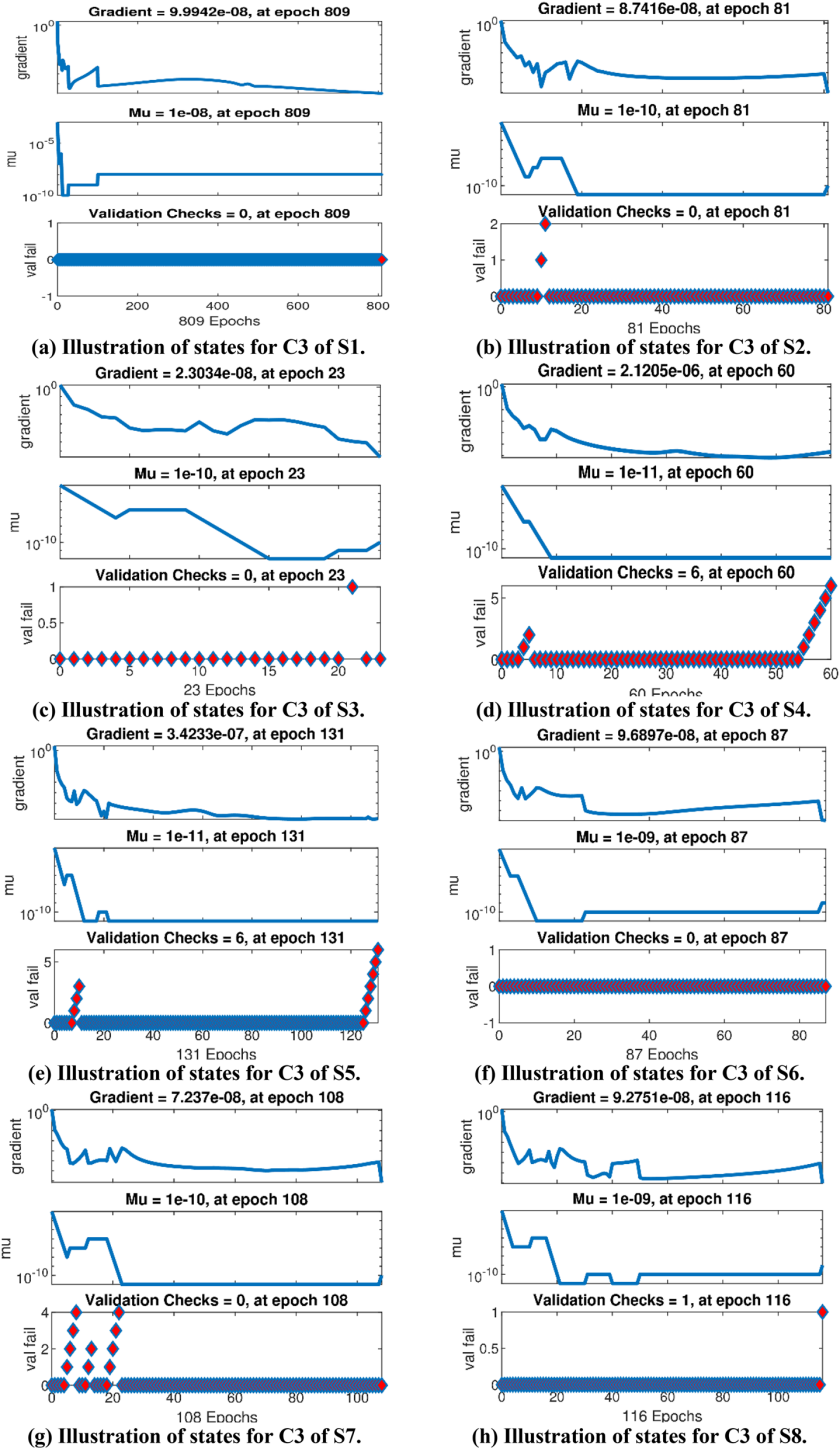
Graphical illustration of Training states of C3 of all eight scenarios.

**Figure 7:**
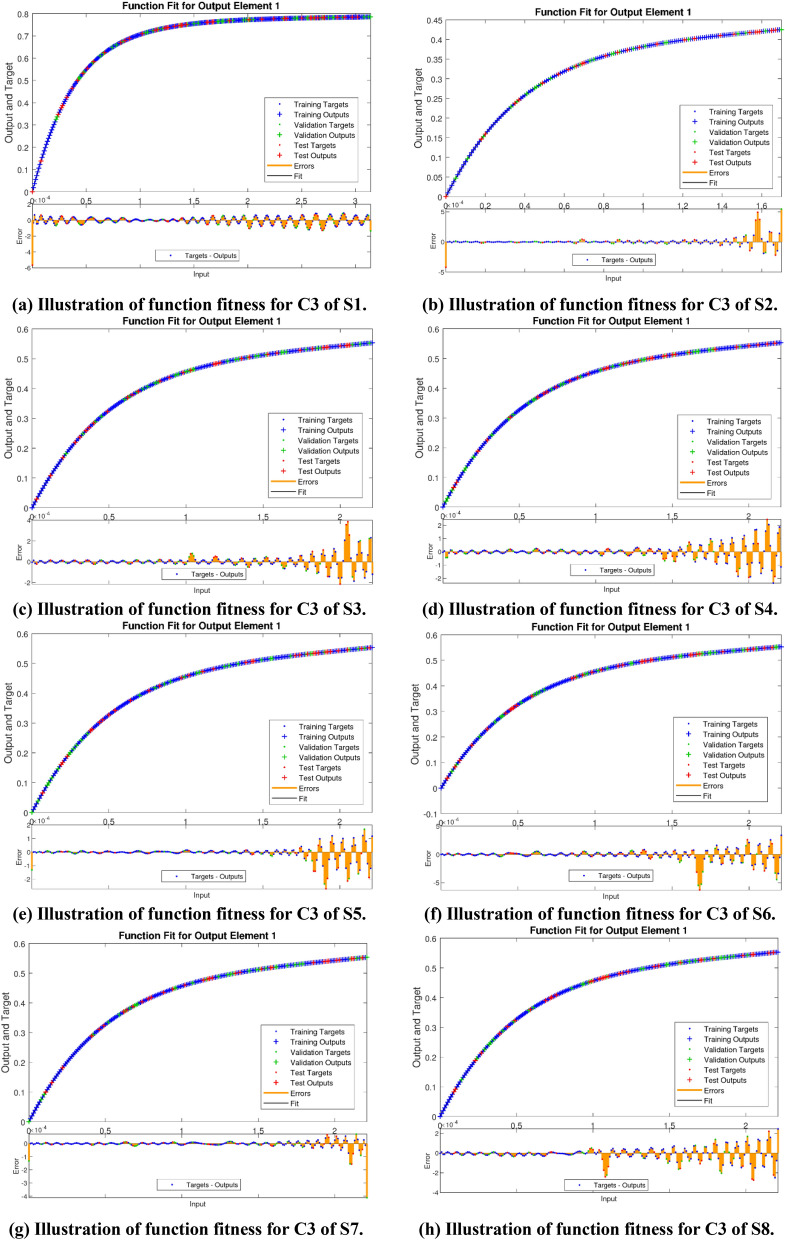
Fitness graphs of C3 of all eight scenarios.

**Figure 8:**
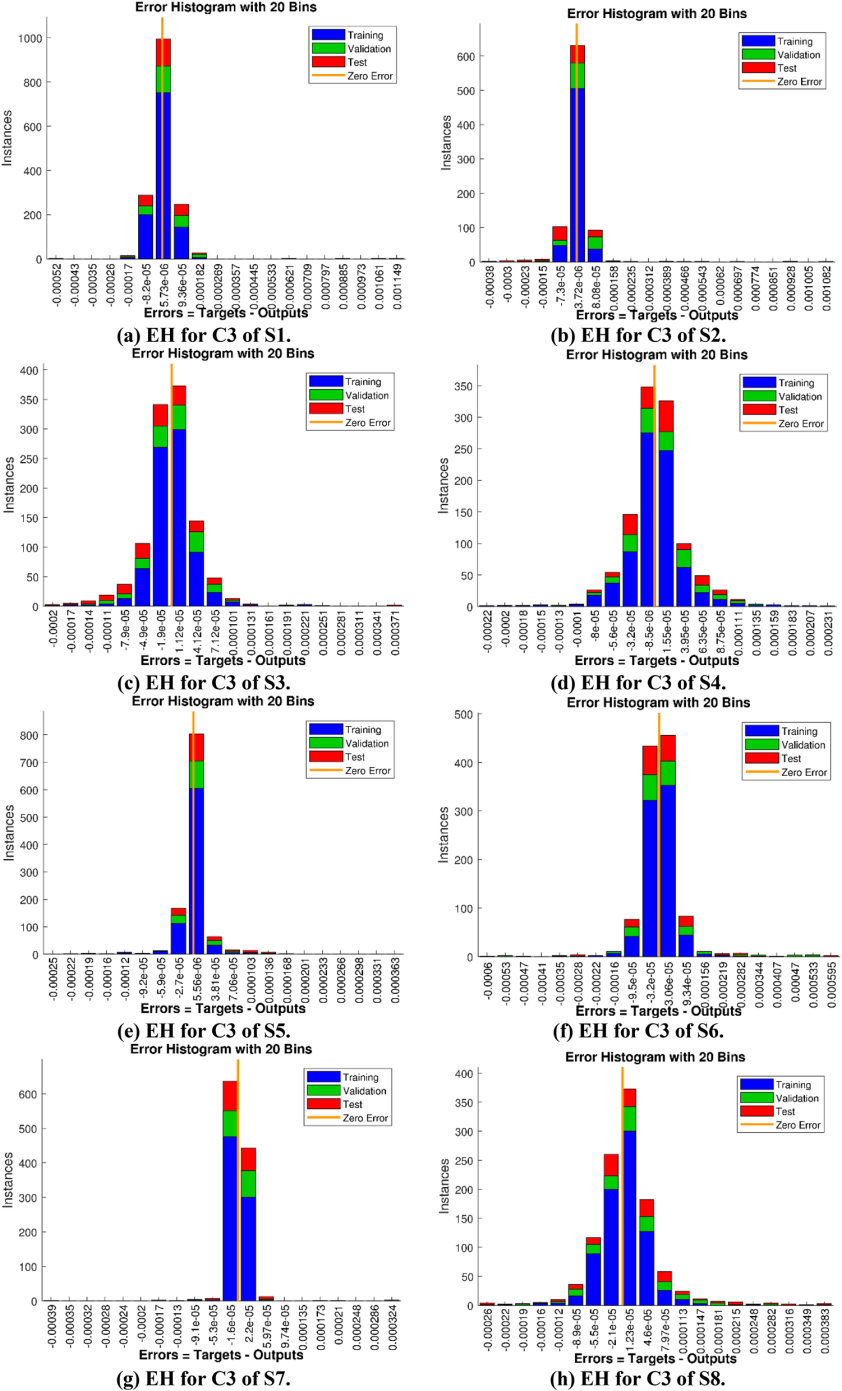
Illistration via Error histograms(EH) for C3 of all eight scenarios.

**Figure 9:**
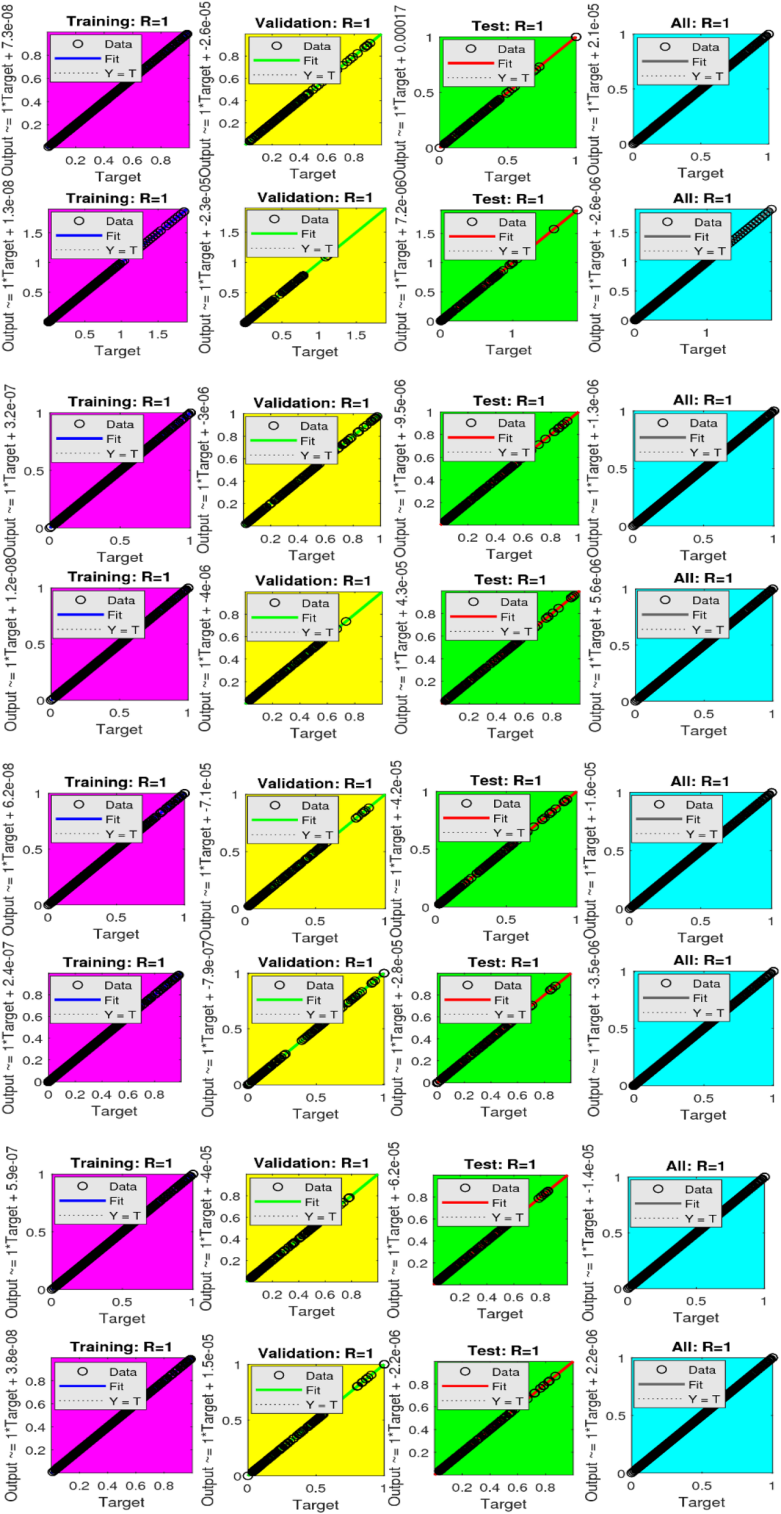
Illustrations via regression plots for C3 of all eight scenarios.

**Figure 10:**
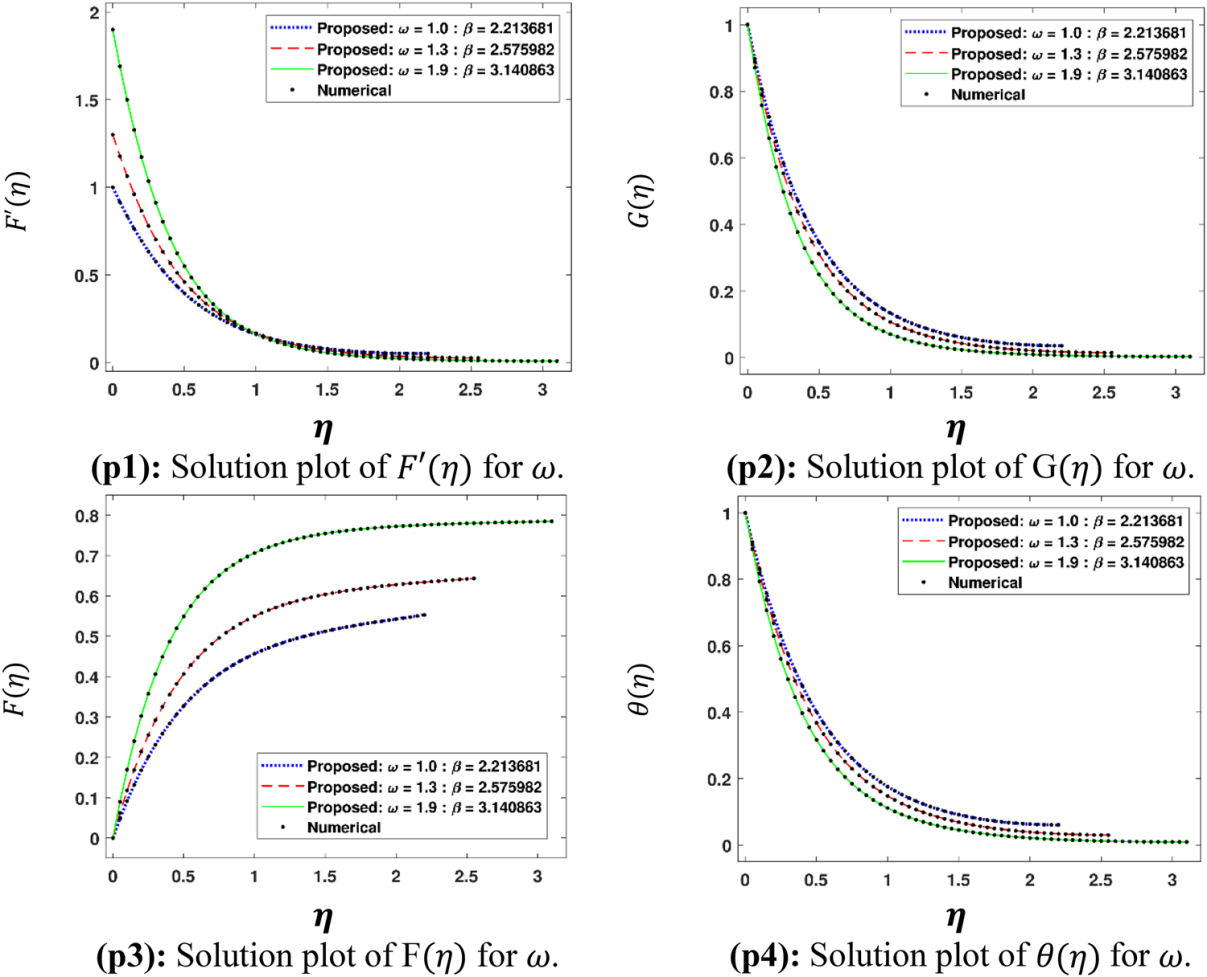
Solution plots of $F^{\prime}\left( \eta \right)$, G $\left( \eta \right)$, F $\left( \eta \right)$ and $\theta \left( \eta \right)$ by using the proposed scheme.

**Figure 11:**
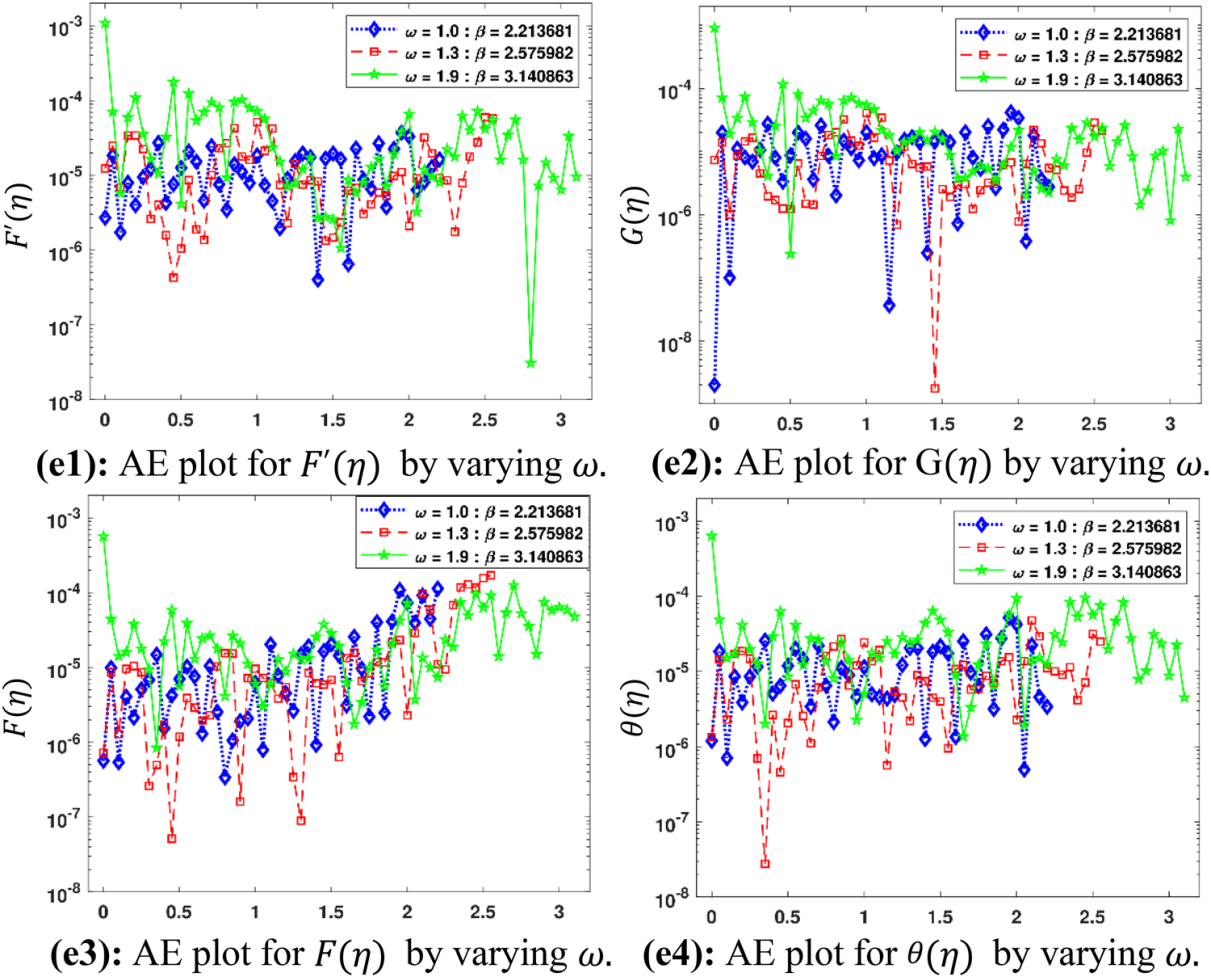
AE plots for outputs by proposed scheme and reference data.

The Figs. 5a–h and 6a–h illustrate the results of case 3 of all the eight scenarios (1–8) showing performance as mean square error (MSE) analysis and the training states detail, respectively. Figure 7a–h explains Fitting graphs for case 3 of scenarios 1–8, respectively. In Fig. 8a–h, the histograms based on error analysis are given, while regression analysis, for each case 3 of all eight scenarios of TFFMFTDECR model, is presented in Fig. 9. The solution graphs are given in Figs. 10p1–p4, 12p5–p8, 14p9–p12 and 16p13–p15, while graphs of absolute error (AE) of the outcomes of the proposed NNs-BLMS and that of the reference solutions are provided in Figs. 11e1–e4, 13e5–e8, 15e9–e12 and 17e13–e15, respectively. Moreover, the Table 4 is provided to illustrate the convergence based on mean square error, epochs, time of execution, performance and the measures of backpropagation scheme for cases 1–3 of scenarios 1–8 for TFFMFTDECR model.

**Table 4:** Various statistics of scenarios (1–8) for cases (1–3) of TFFMFTDECR model.

Scenario	Case	Mean square error			Performance	Gradient	Mu	Epoch	Time
		Training	Validation	Testing					
1	1	3.51E−10	1.58E−9	5.56E−10	3.52E−10	7.06E−8	1.00E−10	96	1
	2	5.16E−10	1.03E−9	6.65E−10	5.16E−10	9.96E−8	1.00E−8	728	17
	3	2.18E−9	4.73E−9	2.14E−8	2.19E−9	9.99E−8	1.00E−8	809	14
2	1	3.49E−11	6.75E−10	1.01E−10	3.5E−8	2.56E−8	1.00E−10	198	3
	2	6.91E−10	7.21E−9	1.06E−8	6.91E−10	9.92E−8	1.00E−11	128	2
	3	9.27E−10	4.93E−9	4.08E−8	9.27E−10	8.74E−8	1.00E−10	81	1
3	1	1.07E−9	5.67E−9	4.59E−9	1.089E−9	8.30E−8	1.00E−10	67	1
	2	2.65E−10	5.35E−10	4.30E−10	7.99E−11	9.47E−8	1.00E−11	38	1
	3	1.25E−9	2.96E−9	6.20E−9	1.25E−9	2.30E−8	1.00E−10	23	1
4	1	7.53E−10	5.92E−9	2.95E−9	7.53E−10	4.41E−8	1.00E−10	86	1
	2	2.59E−10	9.20E−10	1.21E−9	2.07E−10	5.89E−8	1.00E−10	88	1
	3	1.46E−9	2.07E−9	2.44E−9	9.59E−10	2.12E−6	1.00E−11	60	1
5	1	1.29E−9	5.27E−9	4.74E−9	1.30E−9	9.83E−8	1.00E−10	48	1
	2	1.81E−9	6.98E−9	6.67E−9	1.67E−9	9.74E−8	1.00E−10	58	1
	3	4.45E−10	4.08E−9	3.01E−9	3.00E−10	3.20E−7	1.00E−11	131	2
6	1	8.64E−10	2.16E−8	3.30E−9	8.34E−10	1.45E−6	1.00E−9	851	12
	2	2.16E−10	2.80E−10	3.45E−10	9.65E–11	2.78E−6	1.00E−12	44	1
	3	2.54E−9	2.14E−8	1.59E−8	2.55E−9	9.69E−8	1.00E−9	87	1
7	1	1.16E−9	4.49E−9	4.20E−9	9.10E−10	5.39E−7	1.00E−11	63	1
	2	1.21E−9	9.59E−9	5.90E−9	1.22E−9	7.23E−8	1.00E−9	131	2
	3	1.19E−10	3.38E−9	5.45E−10	1.20E−10	7.24E−8	1.00E−10	108	1
8	1	1.74E−9	8.01E−9	1.09E−8	1.66E−9	9.28E−8	1.00E−9	116	1
	2	4.05E−10	2.20E−9	2.40E−9	3.61E−10	2.00E−7	1.00E−11	116	1
	3	1.74E−9	8.01E−9	1.09E−8	1.66E−9	9.28E−8	1.00E−9	116	1

The eight sub-figures (a)–(h) of Fig. 5, gives the convergence of the solution in terms of MSE for train, validation and testing of case 3 of scenarios 1–8 of TFFMFTDECR model. In Table 4, it can be seen that best performance achieved at 809, 81, 23, 60, 131, 87, 108 and 116 epochs with MSE around 10^–9^–10^–8^, 10^–10^–10^–8^, 10^–9^, 10^–9^, 10^–10^–10^–9^, 10^–9^–10^–8^, 10^–10^–10^–9^ and 10^–9^–10^–8^ , respectively. Corresponding values of gradients are [9.9 × 10^–08^, 8.74 × 10^–08^, 2.30 × 10^–08^, 2.12 × 10^–06^, 3.20 × 10^–07^, 9.69 × 10^–08^, 7.24 × 10^–08^ , 9.28 × 10^–08^], while step size $Mu$ for case 3 of all the eight scenarios is [10^–8^, 10^–10^, 10^–10^, 10^–11^, 10^–11^ , 10^–9^, 10^–10^, 10^–09^] and time of execution in seconds for the corresponding case 3 of eight scenarios is [14, 1, 1, 1, 2, 1, 1, 1], which shows the convergence and accuracy of the proposed solver NNs-BLMS.

The states of convergence for case 3 of all eight scenarios are given in subgraphs (a)–(h) of Fig. 6, while the comparison of the results of designed solver NNs-BLMS and the reference solution of the corresponding cases is presented in subgraphs (a)–(h) of Fig. 7. The above mentioned-cases are further studied in the form of error histograms (EH) in subfigures (a)–(h) of Fig. 8. It can be seen that the bins near to desired zero line having values about 5.7 × 10^–06^, 3.7 × 10^–06^, 1.1 × 10^–05^, − 8.5 × 10^–06^, 5.5 × 10^–06^, 3.0 × 10^–05^, − 1.6 × 10^–05^ and 1.2 × 10^–05^. The results of regression analysis are provided graphically for the stated cases. The numerical value of correlation coefficient is consistently nearly equal to 1, for testing, validation and train datasets, which justify the worthy performance of designed solver NNs-BLMS to solve the eight scenarios of TFFMFTDECR model.

**Figure 12:**
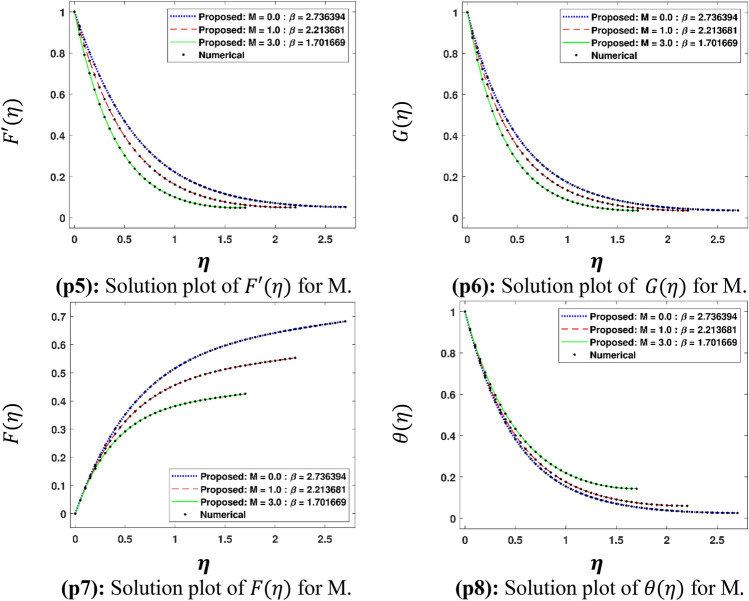
Solution plots of $F^{\prime}\left( \eta \right)$, G $\left( \eta \right)$, F $\left( \eta \right)$ and $\theta \left( \eta \right)$ by using the proposed scheme.

**Figure 13:**
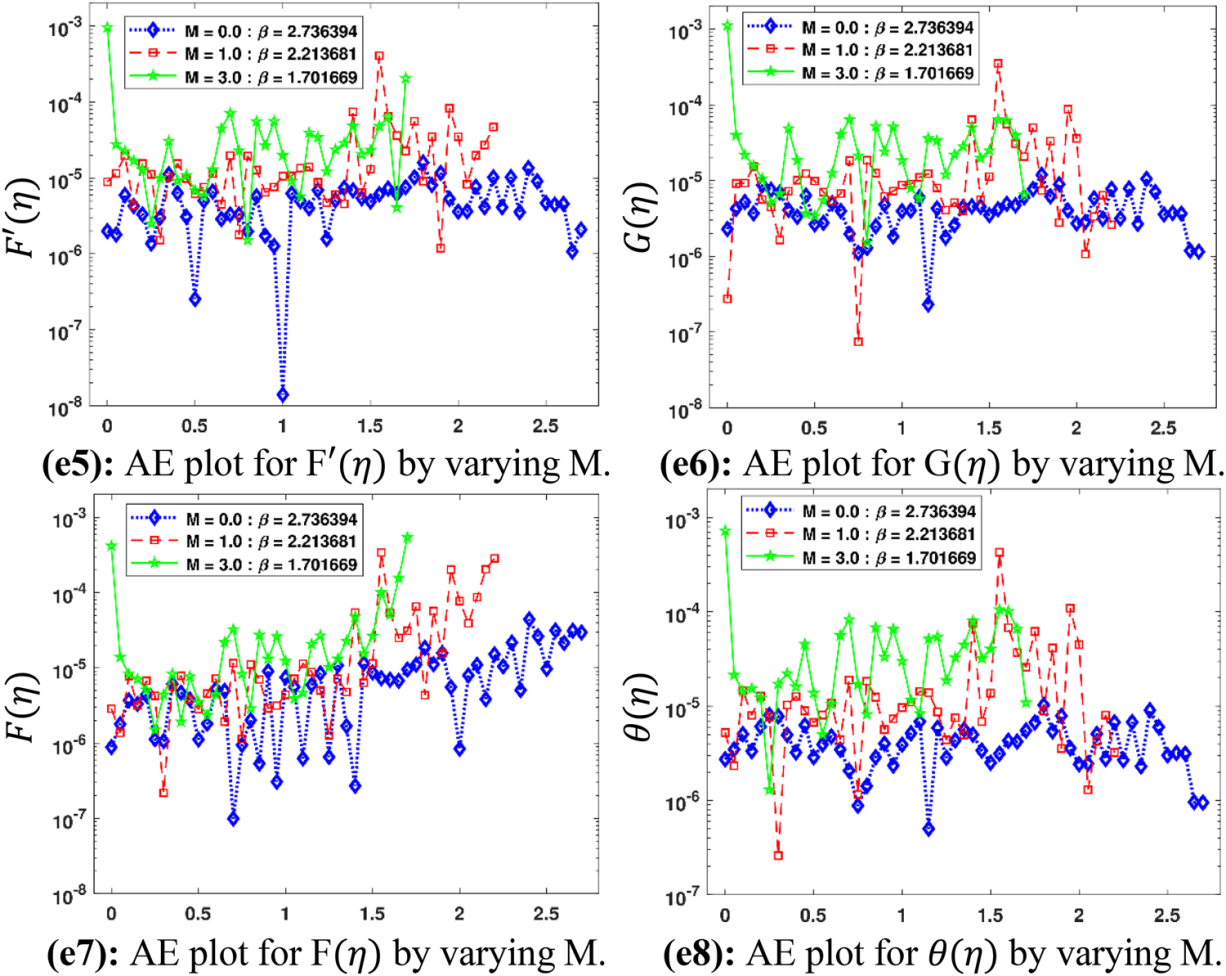
AE plots for outputs by proposed scheme and reference data.

The variation of physical parameters $\omega$, $M$, $A\;( - )$, $B\;( - )$, $A\;( + )$, $B\;( + )$, $\left( {Sr,\;Du} \right)$ and $K_{R}$ represents eight scenarios, where $A\;( - )$ and $B\;( - )$ represents the negative values of parameters $A$ and $B$ respectively. Similarly $A\;( + )$ and $B\;( + )$ the positive values of parameters $A$ and $B$ respectively. The solution plots of velocity component in radial direction $F^{\prime}\left( \eta \right)$, in axial direction $G\left( \eta \right)$, azimuthal direction $F\left( \eta \right)$ and temperature profile $\theta \left( \eta \right)$ are displayed for the variation of $\omega$ i.e. scenario 1 in subplots (p1)–(p4) of Fig. 10, while there corresponding Absolut Error (AE) plots are presented by subplots (e1)–(e4) of Fig. 11. It is obvious from Fig. 10 that $F^{\prime}\left( \eta \right)$ and $F\left( \eta \right)$ increases, while $G\left( \eta \right)$ and $\theta \left( \eta \right)$ decreases for higher $\omega$. The rotation parameter is the ratio of stretching to swirling rates. Thus, the progression in the rotation of disc results a higher stretching behavior in comparison with rotation effect, which causes higher radial flow velocity. In case of moving away from the disc surface, the radial velocity becomes lower. As the stretching effect is dominant at the surface, therefore, moving away from the surface results a decrease in the radial velocity. The decreasing trend of axial velocity component is because of fluid particles, which are moving towards radial direction are compensated, due to rotation, by moving downward at the expense of diminishing axial velocity profile. The results are in line with the published work of Mushtaq and Mustafa^37^. It is depicted in Fig. 11 that AE for $F^{\prime}\left( \eta \right)$, $G\left( \eta \right)$, $F\left( \eta \right)$ and $\theta \left( \eta \right)$ are 10^–7^ to 10^–3^, 10^–8^ to 10^–3^, 10^–8^ to 10^–3^ and 10^–8^ to 10^–3^, respectively. The solution plots of $F^{\prime}\left( \eta \right)$, $G\left( \eta \right)$, $F\left( \eta \right)$ and $\theta \left( \eta \right)$ are shown variation of $M$ i.e. scenario 2 in subplots (p5)–(p8) of Fig. 12, while there corresponding AE plots are given by subplots (e5)–(e8) of Fig. 13. It can be seen from Fig. 12 that $F^{\prime}\left( \eta \right)$, $F\left( \eta \right)$ and $G\left( \eta \right)$ decrease, while $\theta \left( \eta \right)$ enhances for higher $M$. A drag-like force is induced by the implementation of magnetic field in the axial direction. The consequences of this force are in the form of dinishing the velocities in all the directions at the cost of enhancement of the temperature profile. The same results are observed by Maleque^38^. Its is notable that higher magnetic parameter in axial direction enforces the fluid particles a higher resistance, reduces the reduction in fluid velocity and enhances temperature profile of the fluid. It is seen in Fig. 13 that AE for $F^{\prime}\left( \eta \right)$, $G\left( \eta \right)$, $F\left( \eta \right)$ and $\theta \left( \eta \right)$ are 10^–8^ to 10^–3^, 10^–8^ to 10^–3^, 10^–8^ to 10^–3^ and 10^–7^ to 10^–3^, respectively.

**Figure 14:**
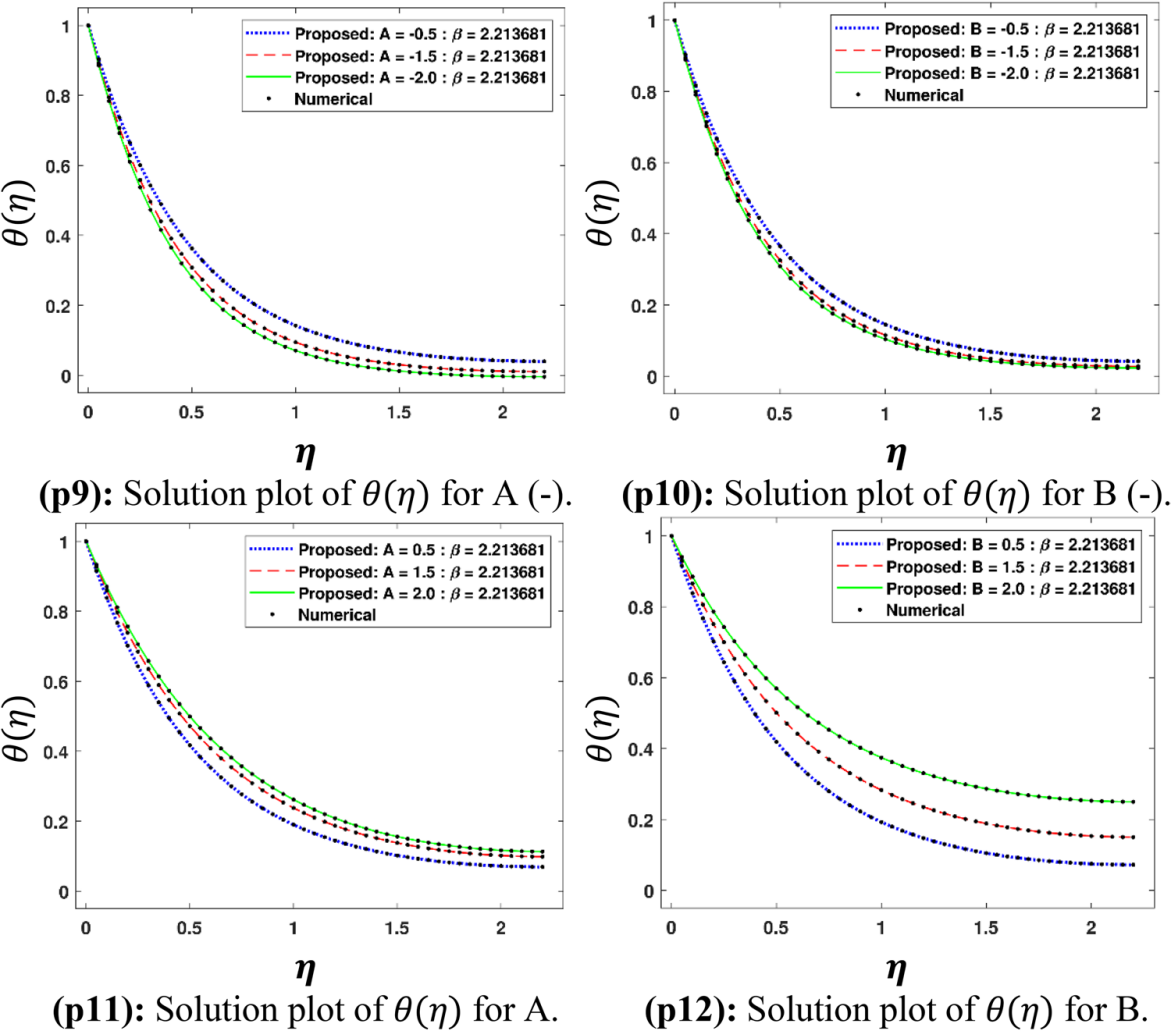
Solution plots of $\theta \left( \eta \right)$ by using the proposed scheme.

**Figure 15:**
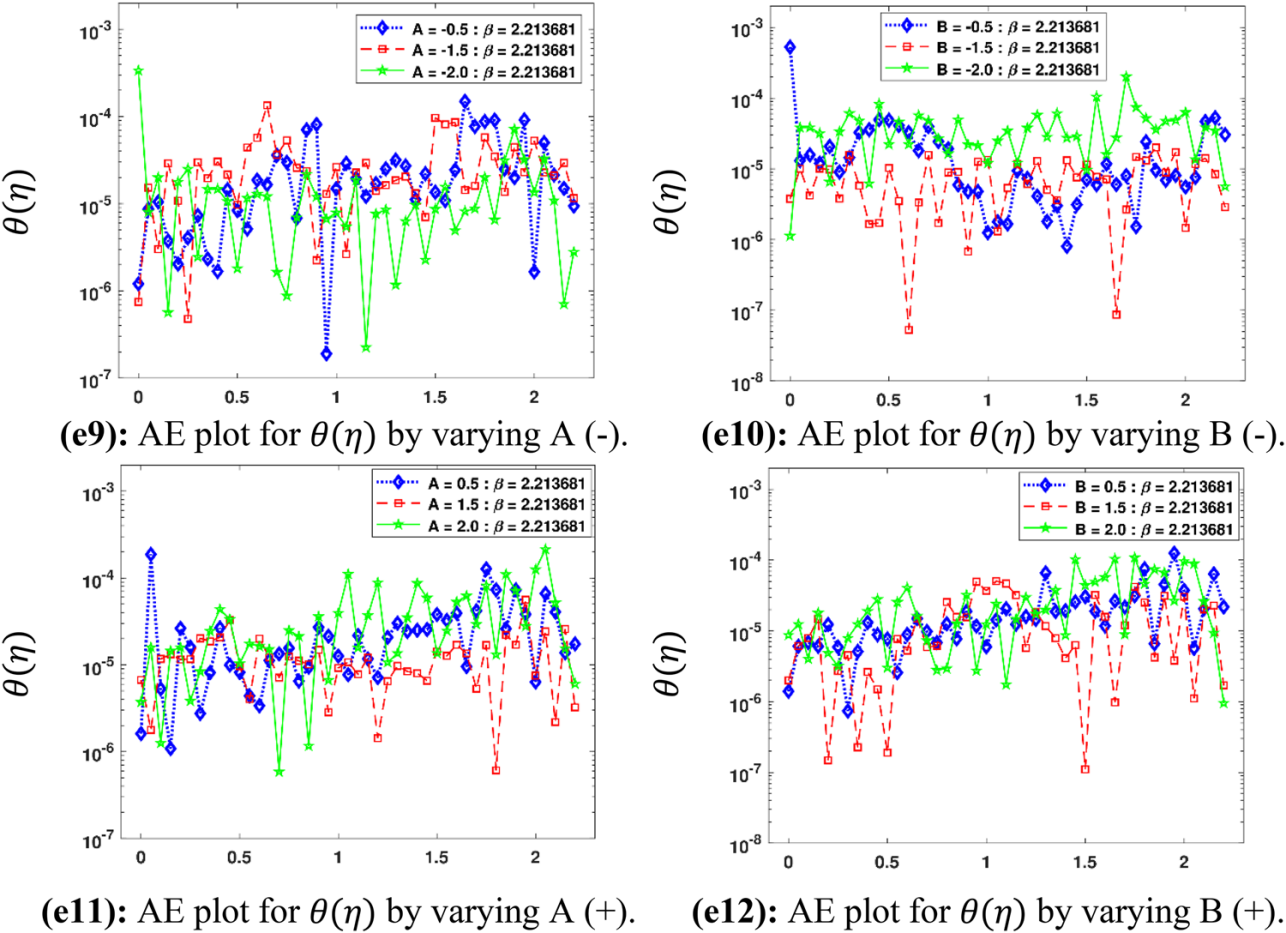
AE plots for outputs by proposed scheme and reference data.

The solution plots of $\theta \left( \eta \right)$ are shown variation of $A\;( - )$, $B\;( - )$, $A\;( + )$ and $B\;( + )$ i.e. scenarios 3–6 in subplots (p9)–(p12) of Fig. 14, while there corresponding AE plots are given by subplots (e9)–(e12) of Fig. 15. It can be seen from Fig. 14 that $\theta \left( \eta \right)$ decreases for decreasing values of $A\;( - )$ and $B\;( - )$, while it increases for higher values of $A\;( + )$ and $B\;( + )$. The temperature/space dependent heat source/sink parameters have considerable effect on the dynamics of temperature profile. The decreasing values of $A\;( - )\;{\text{and}}\;B\;( - )$, which represent the heat sink parameters, cause reduction in temperature profile of the fluid. This result is physically acceptable because it implies absorption of heat in the fluid flow, which consequently, results the reduction in the thickness of boundary layer of Maxwell fluid. On the other hand higher values of $A\;\;{\text{and}}\;B\;$ mean heat generation of space/temperature dependent parameters. The enhancement in these parameters depict the enhancement of the temperature boundary layer thickness, which results mounting in the temperature profile. The results of this study have an agreement with the outcomes obtained by Irfan et al. ^31^. It is seen in Fig. 15 that AE for $\theta \left( \eta \right)$ are 10^–7^ to 10^–3^, 10^–8^ to 10^–3^, 10^–7^ to 10^–3^ and 10^–8^ to 10^–3^, corresponding to $A\;( - )$, $B\;( - )$, $A\;( + )$ and $B\;( + )$, respectively.

**Figure 16:**
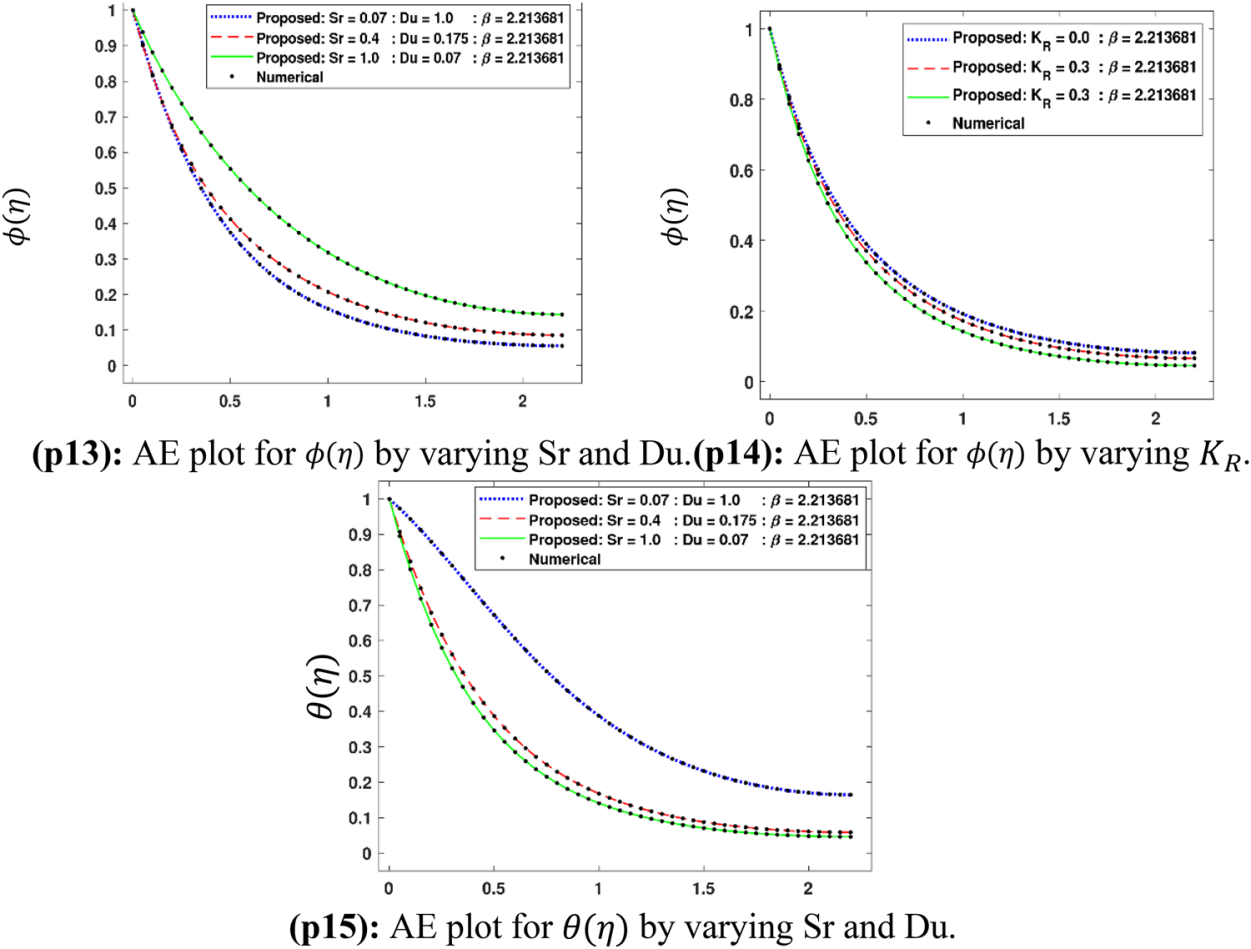
Solution plots of $\theta \left( \eta \right)$ and $\phi \left( \eta \right)$ by using the proposed scheme.

**Figure 17:**
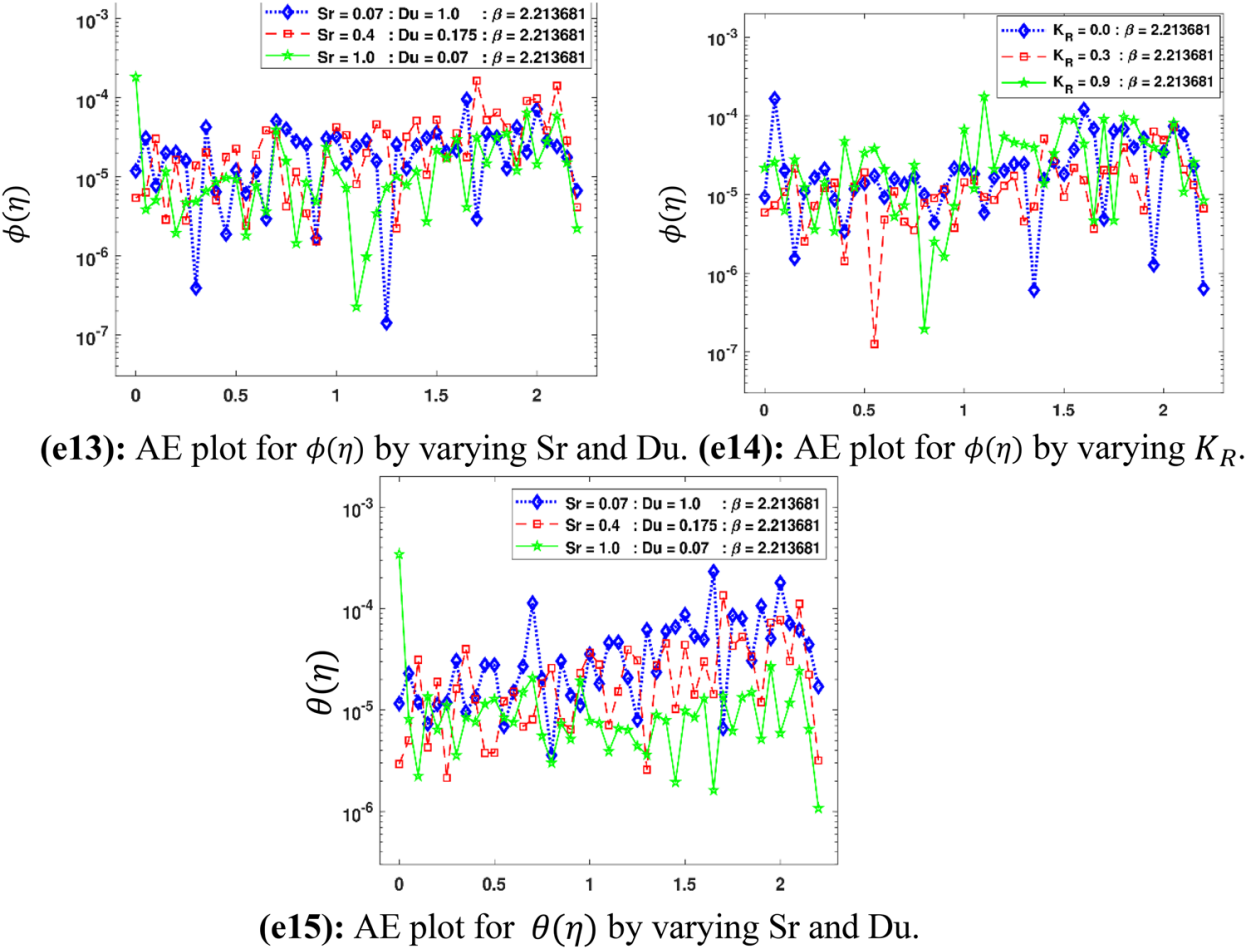
AE plots for outputs by proposed scheme and reference data.

The solution plots of $\phi \left( \eta \right)$, $\theta \left( \eta \right)$ for increasing Soret number $Sr$ and consequently decreasing Dufour $Du$ number, while the solution graph of $\phi \left( \eta \right)$ for increasing values of reaction rate parameter $K_{R}$ are presented in subgraphs (p13)–(p15) of Fig. 16, whereas the corresponding error graphs are provided in subgraphs (e13)–(e15) of Fig. 17. The stated variation of $Sr$ and $Du$ causes an increase in concentration profile, while a decrease in temperature profile (see Fig. 16p13, p15), whereas the concentration profile diminishes for the stated variation of $K_{R}$ (see Fig. 16p14). The boosting chemical reaction rate has influence on concentration profile to reduce because of the reason that during chemical reaction, the consumption of chemical species takes place, which causes the reduction in solutal concentration profile. The mass flux caused by a temperature difference termed as Soret effect, while the energy flux due to concentration gradient is called Dufour effect. The higher Soret and diminishing Dufour effects causes the higher concentration boundary layer thickness of the fluid. This is because of the higher mass flux causes an increase in the concentration profile. A declining trend in temperature profile is seen for higher Soret and simultaneous declining Dufour number. This is due to decreasing temperature flux due to less concentration difference. The AE plots Fig. 17e13,e15 have AE 10^–7^ to 10^–3^ and 10^–6^ to 10^–3^, while Fig. 17e14 have AE of 10^–6^ to 10^–3^.

## Conclusions

The designed numerical computing paradigm through intelligent neural networks with backpropagation scheme of Levenberg–Marquardt, is applied to solve the Maxwell nanofluid model of thin film flow with thermo-diffusion effect and chemical reaction. The reference dataset for NNs-BLMS was taken by using Adams numerical solver to solve TFFMFTDECR system in case of eight variants based on rotation parameter, magnetic parameter, space dependent heat source/sink, heat dependent heat source/sink, combine effects of Soret and Dufour numbers, and reaction rate parameter. The 70% data was used for train, 15% for validation and 15% for testing processes through NNs-BLMS and matching level of accuracy around 10^–11^ to 10^–3^ between the proposed and reference solutions show the precision and consistency. The performance validated through inferences from presented numerical tables, graphical descriptions of convergence plots, MSE, regression illustrations and error-histogram analyses. The outcomes of physical parameters of interest for TFFMFTDECR model revealed that:

- The decreasing trend of axial velocity component is because of fluid particles, which are moving towards radial direction are compensated, due to rotation, by moving downward at the expense of diminishing axial velocity profile.
- The higher rotation parameter enhances fluid film thickness and diminishes the temperature profile.
- Higher magnetic field diminishes liquid film thickness, while enhances temperature profile.
- Higher Soret number and corresponding diminishing Dufour number enhances the concentration profile, while declining trend in temperature profile is seen for higher Soret and simultaneous declining Dufour number. This is due to decreasing temperature flux due to less concentration difference.
- Higher reaction rate parameter causes decreasing concentration profile.

## List of symbols

(*u*, *v*, *w*): Components of velocity
NNs: Neural Networks
$$\left( {r,\;\phi ,\;z} \right)$$: Cylindrical coordinates
BLM: Backpropagated Levenberg-Marquardt scheme
$\upsilon ,\;\mu$: Kinematic and dynamic viscosities
h: Thin film thickness
$\lambda_{1}$: Relaxation parameter of time
*k*: Thermal conductivity
*t*: Time
*T*, *C*: Temperature and concentration of fluid
$C_{S} ,T_{S}$: Concentration and temperature at surface
$T_{ref} ,C_{ref}$: Constant reference temperature and concentration
$C_{0} ,T_{0}$: Concentration and temperature at origin
*B*: Time dependent magnetic parameter
$\Omega$: The disk rotation rate
c: The stretching rate of the disk
$q^{\prime\prime\prime}$: Heat source/sink
a: Positive constant
*D*_*T*_: Coefficient of thermophoresis diffusion
$$B_{0}$$: Magnetic parameter
*D*_*B*_: Brownian diffusion
$\rho$: Density of the fluid
$$K_{T}$$: Thermal diffusion
${c}_{p}$: Specific heat subjected to constant pressure
*ɳ*: Dimensionless variable
${\text{Nu}}_{r}$: Local Nusselt number
$$k_{V}$$: Thermophoretic coefficient
kr: Chemical reaction coefficient
$T_{m}$: Mean temperature of fluid
Pr: Prandtl number
*S*: Unsteadiness parameter
*τ*: Thermophoretic parameter
${\text{Sh}}_{r}$: Local Sherwood number
*S*: Unsteadiness parameter
${\text{Re}}$: Local Reynolds number
$G\left( \eta \right)$: Dimensionless velocity in axial direction
$F\left( \eta \right)$: Dimensionless velocity azimuthal direction
$$\phi \left( \eta \right)$$: Dimensionless concentration
$F^{\prime}\left( \eta \right)$: Dimensionless velocity in radial direction
$$\theta \left( \eta \right)$$: Dimensionless temperature
$K_{R}$: Chemical reaction parameter
*Sc*: Schmidt number
$$\beta_{1}$$: Deborah number
$\beta$: Value of $\eta$ at free surface
$\omega$: Dimensionless rotation parameter
$M$: Dimensionless magnetic parameter
$B^{*}$: Heat source/sink depending on temperature
*Du*: Dufour number
*Sr*: Soret number
$$V_{T}$$: Thermophoretic velocity
$A^{*}$: Space dependent heat source/sink
